# Cutaneous Deterioration Following Glucocorticoid Discontinuation in Psoriatic Arthritis: A Real-World Cohort Study

**DOI:** 10.3390/jcm15197359

**Published:** 2026-09-22

**Authors:** Clara Gratton, Boris Ehrenstein, Florian Günther, Louisa Schminke, Martin Fleck, Olga B. Krammer

**Affiliations:** 1 Faculty of Medicine, University of Regensburg, 93053 Regensburg, Germany; 2 Department of Rheumatology and Clinical Immunology, Asklepios Hospital Bad Abbach, 93077 Bad Abbach, Germany; 3 Centre for Rheumatologic Rehabilitation, Asklepios Hospital Bad Abbach, 93077 Bad Abbach, Germany; 4 Department of Internal Medicine I, University Medical Centre, 93053 Regensburg, Germany

**Keywords:** arthritis, psoriatic, glucocorticoids, psoriasis, drug tapering, cohort studies, retrospective studies

## Abstract

**Background/Objectives:** To evaluate the frequency of documented cutaneous deterioration within three months after complete discontinuation of systemic glucocorticoid therapy in psoriatic arthritis (PsA) and to assess a potential association with cumulative glucocorticoid exposure. **Methods:** This retrospective single-centre cohort study included 100 patients with PsA fulfilling CASPAR criteria who received systemic glucocorticoids at a tertiary rheumatology centre between 2008 and 2024. The primary endpoint was the occurrence of cutaneous deterioration within three months after complete glucocorticoid discontinuation. Associations with cumulative prednisolone-equivalent dose were analysed using logistic regression. **Results:** Cutaneous deterioration occurred in 10% of patients within three months after glucocorticoid discontinuation. Only a minority of cases were clinically pronounced; no erythrodermic or generalized pustular flares were observed. No statistically significant association between cumulative glucocorticoid exposure and cutaneous deterioration was found (OR 0.90; 95% CI 0.67–1.22). **Conclusions:** In this real-world PsA cohort, documented cutaneous deterioration following glucocorticoid discontinuation was infrequent and predominantly mild. No statistically significant association between cumulative glucocorticoid exposure and cutaneous deterioration was detected. However, due to the absence of a control group, no conclusions can be drawn regarding a causal relationship between glucocorticoid discontinuation and subsequent skin deterioration.

## 1. Introduction

Psoriasis is a chronic immune-mediated inflammatory disease primarily affecting the skin. Approximately 30% of patients with psoriasis develop psoriatic arthritis (PsA) during their disease course [1,2]. Psoriatic arthritis is a systemic inflammatory disease affecting peripheral and axial joints and is driven by immune pathways including the IL-23/IL-17 axis [3]. Due to its inflammatory nature, systemic glucocorticoids would theoretically be effective in controlling disease activity. However, their use in psoriasis and PsA has historically been approached with caution due to concerns about potential cutaneous exacerbations, particularly during dose tapering or withdrawal.

These concerns originate from early case reports and series from the 1960s, which suggested an association between systemic glucocorticoids and severe psoriasis flares, including pustular transformation [4,5]. Therefore, clinical guidelines have long recommended cautious use and gradual tapering of systemic glucocorticoids. GRAPPA (Group for Research and Assessment of Psoriasis and Psoriatic Arthritis) guidelines initially recommended only short-term use at low doses with careful tapering to minimize potential flares [6].

However, the evidence supporting this long-standing concern remains limited. Recent systematic analyses report low rates of dermatological flares under or after glucocorticoid therapy. Vincken et al. analyzed 21 studies including more than 4.1 million patients and found flare rates between 0% and 1.42% [7], suggesting that the true risk may be lower than historically assumed. Accordingly, the most recent EULAR (European Alliance of Associations for Rheumatology) 2023 recommendations no longer explicitly address systemic glucocorticoids, citing limited evidence and safety concerns [8], whereas EuroGuiDerm continues to recommend cautious tapering despite the lack of robust supporting data [9].

Thus, current guideline recommendations remain inconsistent, and the clinical relevance and frequency of dermatological deterioration following glucocorticoid tapering in PsA remain insufficiently defined.

Potential associations between cumulative glucocorticoid exposure or treatment duration and the risk of cutaneous deterioration have not been systematically evaluated. Moreover, prospective data on tapering strategies and real-world cutaneous outcomes in rheumatological routine care are lacking.

Therefore, the primary aim of this study was to determine the frequency and clinical characteristics of documented cutaneous deterioration within three months after glucocorticoid discontinuation in patients with psoriatic arthritis. As a secondary objective, we explored whether cumulative glucocorticoid exposure was associated with the occurrence of cutaneous deterioration.

## 2. Materials and Methods

This retrospective, single-centre cohort study was conducted at a tertiary rheumatology centre. It included patients who were seen in either an inpatient or outpatient setting between 2008 and 2024. A total of 1611 patients were screened for eligibility. Of these, 100 fulfilled the predefined inclusion criteria and were included in the final analysis.

Only patients who met the Classification Criteria for Psoriatic Arthritis (CASPAR) from 2006 [10] were included. In addition, all patients had a confirmed dermatological diagnosis of psoriasis vulgaris, pustular psoriasis, erythrodermic psoriasis, or nail psoriasis. Patients were excluded in case of a suspected but unconfirmed diagnosis of psoriatic arthritis (not fulfilling CASPAR criteria), absence of a documented dermatological diagnosis of psoriasis, coexisting inflammatory rheumatic diseases (e.g., rheumatoid arthritis or systemic lupus erythematosus), or incomplete documentation of glucocorticoid therapy or clinical course. All included patients had received systemic glucocorticoid therapy during their inpatient stay or outpatient visit. Dose, duration, and cumulative exposure varied between individuals and were recorded separately for analysis. Following complete tapering of glucocorticoid therapy, the subsequent three-month period was systematically assessed for dermatological deterioration (Figure 1).

Data were collected from medical records and included demographic characteristics, comorbidities, clinical manifestations of psoriatic arthritis and psoriasis, laboratory inflammatory markers (CRP), details of systemic glucocorticoid therapy (preparation, starting dose, duration, cumulative prednisolone-equivalent dose), concomitant antirheumatic treatment, and glucocorticoid-related adverse events.

As Psoriasis Area and Severity Index (PASI) or body surface area (BSA) was not routinely documented, a predefined composite outcome was used to identify documented cutaneous deterioration following glucocorticoid discontinuation. Cutaneous deterioration was considered present if at least one of the following criteria was fulfilled within three months after complete glucocorticoid discontinuation:

- a physician-documented increase in psoriasis symptoms [7,11,12].
- the appearance of new or extended psoriatic skin lesions [13,14,15].
- treatment escalation due to skin symptoms [12,13].
- dermatological consultation or treatment because of a worsening of psoriasis [11,12].

The local ethics committee responsible for our institution approved the study (approval code 12-101-0074, approval date 21 June 2012), and patients provided written consent for the analysis of anonymized data for scientific purposes. As a retrospective observational cohort study, this study was not registered in a clinical trial registry (not applicable). IBM SPSS Statistics, version 29.0.2.0 (IBM Corp., Armonk, NY, USA) was used for the statistical analysis. Data analysis was initially performed at a descriptive level. Continuous variables (e.g., age, BMI, CRP) are presented as mean ± standard deviation in case of normal distribution or as median [IQR], while categorical variables are reported as absolute and relative frequencies. Exploratory comparisons were performed between patients with and without documented cutaneous deterioration. Continuous variables were compared using the Mann–Whitney U test, while the chi-square test or Fisher’s exact test was applied for categorical variables. In addition, a univariate logistic regression analysis was performed to assess the association between cumulative glucocorticoid dose and documented cutaneous deterioration (odds ratio, 95% CI, *p*-value).

## 3. Results

### 3.1. Baseline Characteristics

We included 100 adults with psoriatic arthritis fulfilling the CASPAR criteria and treated with systemic glucocorticoids at a tertiary rheumatology centre between 2008 and 2024. Of these patients, 58% (*n* = 58) were admitted as inpatients and 42% (*n* = 42) presented as outpatients. The cohort was predominantly male (60% vs. 40%), with a mean age of 53.5 years (±SD 13.6). The median body mass index (BMI; with interquartile range, IQR) was 27.0 kg/m^2^ (25.1–30.8 kg/m^2^). Among comorbidities, arterial hypertension was most prevalent (36%), followed by diabetes mellitus and coronary heart disease (each 11%) and hyperlipidaemia (6%). Less frequent comorbidities included renal insufficiency in 6 patients (6%) and inflammatory bowel disease in 2 patients (2%). Active smoking was documented as a cardiovascular risk factor in 12% of the cohort. The baseline characteristics are summarized in Table 1.

Monoarthritis, oligoarthritis, and polyarthritis were present in 25%, 35%, and 40% of patients, respectively. Axial involvement was observed in 17%. Extra-articular manifestations included dactylitis in 16% and enthesitis in 7%. Regarding the dermatological phenotype, the majority of patients (89%) had psoriasis vulgaris, while 9% presented with pustular psoriasis and 2% with isolated nail psoriasis. At presentation, active cutaneous psoriasis was documented in 65% of patients, while nail involvement, irrespective of the underlying psoriasis subtype, was present in 14%.

At the index visit, the median CRP concentration was 9.2 mg/L (IQR 2.9–21.9). At the index visit, the majority of the cohort (61%) received conventional synthetic DMARD therapy, 24% received biological therapy, 13% NSAIDs only and 2% JAK-Inhibitors. 14% of patients received methotrexate as an add-on therapy. 52% of patients had received systemic glucocorticoid therapy prior to the index visit; during the outpatient consultation or inpatient stay, 18% received an intra-articular triamcinolone injection. The median glucocorticoid-starting dose was 12.5 mg (IQR 6.0–20.0); the median duration of therapy was 16.0 weeks (IQR 10.0–50.25). The median cumulative glucocorticoid dose was 729.75 mg prednisolone equivalent (IQR 354.38–1892.25).

### 3.2. Dermatological Course and Side Effects

Within the observation period of three months after complete glucocorticoid discontinuation, a dermatological deterioration was documented in 10 of 100 patients (10%). A detailed assessment of the ten documented cutaneous deteriorations revealed marked heterogeneity in clinical severity. Two patients experienced pronounced exacerbations with a progressive course, whereas the remaining eight events were predominantly described as mild, discrete, or locally limited psoriatic skin lesions. Escalation of systemic therapy due to cutaneous worsening was required in two cases (dose escalation of subcutaneous methotrexate from 15 mg/week to 20 mg/week, and switch from adalimumab to infliximab in another) and intensification of topical treatment in one additional case. No cases of erythrodermic or generalized pustular psoriasis were observed in this cohort. Side effects were documented in 4 of 100 patients (4%) and included weight gain (*n* = 2), sleep disturbance (*n* = 1), and osteoporosis (*n* = 1). However, as the assessment period was limited to three months, long-term adverse effects could not be reliably evaluated.

### 3.3. Exploratory Comparison Between Patients with and Without Dermatological Deterioration

Table 2 summarizes exploratory comparisons between patients with (*n* = 10) and without (*n* = 90) cutaneous deterioration across a broad range of potential associated factors, including demographic characteristics, smoking status and comorbidities, musculoskeletal and dermatological manifestations, background antirheumatic therapy, inflammatory markers, and glucocorticoid treatment characteristics. None of the examined variables differed significantly between the groups. The largest numerical differences were observed for axial manifestations (40.0% vs. 14.4%; *p* = 0.064) and female sex (70.0% vs. 36.7%; *p* = 0.084), both of which were more frequent among patients with cutaneous deterioration, although neither reached statistical significance. No statistically significant differences were observed for smoking status, BMI, the assessed comorbidities, background antirheumatic therapy, CRP, glucocorticoid starting dose, treatment duration, or cumulative glucocorticoid exposure. The median cumulative glucocorticoid dose was 1052 mg [IQR 393.5–1478.2] in patients with cutaneous deterioration and 712.2 mg [IQR 351.9–1945.5] in those without deterioration.

In the univariate logistic regression analysis, cumulative glucocorticoid dose was not statistically significantly associated with cutaneous deterioration (OR 0.90; 95% CI 0.67–1.22; *p* = 0.511).

## 4. Discussion

The present investigation revealed that within three months after discontinuation of systemic glucocorticoid therapy, a dermatological deterioration was documented in 10% of PsA patients. As a secondary finding, no statistically significant association was demonstrated between cumulative glucocorticoid exposure and the occurrence of a dermatological deterioration. Exploratory comparisons did not identify statistically significant factors associated with cutaneous deterioration. With only 10 cutaneous deterioration events, reliable identification of independent predictive factors was not possible, and multivariable modelling was therefore not performed. The frequency of documented cutaneous deterioration (10%) observed within three months after glucocorticoid discontinuation lies considerably above the values reported in several previous studies: Bayrak and Aktas (2024) found, in a treatment-naive PsA cohort with CASPAR diagnosis, no single skin flare after glucocorticoid tapering during a two-year follow-up [16]. Gregoire et al. (2021) reported a rate of 1.42% in a dermatological psoriasis cohort with an identical three-month observation window [14]. In interventional and observational studies that captured cutaneous deterioration during ongoing glucocorticoid therapy, flare rates also remained predominantly below 5% (0% in Babino 2016 [17], Haroon 2018 [18], Saviola 2007 [19], and Carubbi 2016 [20]; in the PASI-based subanalysis by Coates and Helliwell 2016 [21], in which a flare was defined as a PASI increase of ≥2 points from baseline, this criterion was met by 10 of 120 patients (8.3%)).

Importantly, the authors of this trial explicitly note that none of these ten PASI increases translated into a clinically manifest flare: not one was reported as an adverse event, and none triggered a treatment adjustment, indicating a relevant disconnect between an objective PASI change and a clinically apparent worsening of psoriasis. This observation is in line with our own results, in which the rate of clinically pronounced, treatment-requiring cutaneous deteriorations was substantially lower than the overall deterioration rate based on chart documentation.

A considerably higher rate was reported in a small controlled cohort study of 32 psoriasis patients [15]; however, this study is only of limited comparability with a PsA cohort due to its small sample size, exclusively dermatological population, and methodological objections concerning control group selection, possible confounders, and the chosen flare definition [22].

Several factors may contribute to the higher frequency of documented cutaneous deterioration in the present cohort: First, the patient population differs. The Rheumatology Clinic Bad Abbach is a specialized tertiary centre in which PsA patients with complex, active, and treatment-refractory courses are managed, whereas most comparison studies include patients from general rheumatological settings [14,16]. Second, the present investigation reflects real-world conditions, whereas several comparison studies were conducted as randomized controlled trials [19,20], often with extensive concomitant DMARD or biological therapy (Babino 2016: Etanercept [17]; Haroon 2018: 60% DMARDs [18]; Carubbi 2016: 100% DMARDs/biologics [20]), which may partly contribute to differences in dermatological outcomes between studies. Third, the case definition of flares differs: while Coates [21] uses PASI-based criteria, the present study is based on dermatological chart documentation, which captures any clinically observed or subjectively reported deterioration of the skin as an event. This case definition is comparatively broader than PASI-based criteria, as it also includes mild skin changes below a quantitative threshold. It is therefore plausible that the 10% frequency of documented cutaneous deterioration observed in the present study also includes milder or subjectively reported deteriorations that would not have been classified as flares in PASI-based studies. The rate of clinically pronounced, severe exacerbations is therefore likely to be substantially lower.

Notably, most cases of documented cutaneous deterioration were mild, with only a small proportion requiring treatment escalation and no severe forms such as erythrodermic or generalized pustular psoriasis observed. The rate of clinically pronounced, treatment-requiring dermatological deteriorations is approximately 2% and thus of the same order of magnitude as the values reported in PASI-based comparison studies (Gregoire et al. 2021: 1.42%) [14].

The observed cutaneous deterioration should also be interpreted in the context of the naturally fluctuating course of psoriatic disease. In a cohort of patients with moderate-to-severe psoriasis, patients identified as experiencing flares had a median of two flare episodes during the preceding 12 months [23]. Thus, in the absence of a control group, some of the deterioration observed during follow-up may reflect the natural disease course rather than an effect attributable to glucocorticoid discontinuation. The low rate of clinically significant dermatological deterioration (~2%) and the absence of a statistically significant association between cumulative glucocorticoid exposure and cutaneous deterioration observed in the present cohort are consistent with recent systematic reviews and cohort data [7,16,24]. These findings suggest that clinically relevant cutaneous deterioration following glucocorticoid discontinuation was uncommon in this cohort. However, due to the retrospective design and the absence of a control group, no conclusions can be drawn regarding a causal relationship between glucocorticoid discontinuation and subsequent skin deterioration. Accordingly, the present findings should not be interpreted as evidence for the safety of systemic glucocorticoid therapy or as support for a specific glucocorticoid treatment or tapering strategy.

From a clinical perspective, the present study provides real-world data on the frequency and severity of documented cutaneous deterioration following glucocorticoid discontinuation in PsA. These findings may help clinicians contextualize the observed frequency and severity of such events when counselling patients and planning follow-up, while underscoring the importance of structured dermatological monitoring using standardized instruments such as PASI, particularly in patients with active or fluctuating skin disease.

A strength of this study is the well-defined PsA cohort, with all patients fulfilling CASPAR criteria and being managed in a specialized centre for inflammatory rheumatic diseases. Compared with large database studies relying on ICD codes or self-reported diagnoses [7], this approach reduces the risk of diagnostic misclassification. The study is based on real-world clinical data and therefore reflects routine care of PsA patients receiving glucocorticoid therapy. Given the limited availability of randomized controlled trials in this field, it provides additional real-world data on a guideline topic that remains insufficiently supported by high-quality evidence. In contrast to many previous studies that categorize glucocorticoid exposure dichotomously (yes/no), this work uses cumulative prednisolone-equivalent dose as a quantitative exposure measure. This enables a more differentiated assessment of the association between cumulative glucocorticoid exposure and cutaneous deterioration compared with studies that classify glucocorticoid exposure only dichotomously.

However, the present study has several limitations that need to be considered when interpreting the results. First, its retrospective single-centre design limits generalizability to other care settings. Furthermore, the absence of a control group precludes determining whether cutaneous deterioration observed during follow-up was attributable to glucocorticoid discontinuation or reflected the natural course of psoriatic disease. In addition, the relatively small cohort size and the low number of cutaneous deterioration events (*n* = 10) restricted statistical power and precluded multivariable adjustment. Accordingly, subgroup comparisons should be considered exploratory and hypothesis-generating rather than confirmatory. Consequently, a clinically relevant effect of cumulative glucocorticoid exposure cannot be definitively excluded, as reflected by the wide confidence interval (0.67–1.22). Furthermore, the requirement for complete glucocorticoid discontinuation and complete three-month follow-up documentation may have introduced selection bias, as patients with ongoing glucocorticoid therapy or incomplete follow-up were not included in the analysis. Concomitant changes in background antirheumatic therapy during the observation period may have influenced cutaneous outcomes and could not be accounted for in the present analyses.

A further limitation relates to the definition of cutaneous deterioration. Since standardized dermatological outcome measures such as the Psoriasis Area and Severity Index (PASI) or body surface area (BSA) were not routinely documented at our study centre, cutaneous deterioration was identified using a predefined composite outcome based on retrospective chart review. Furthermore, outcome assessment was not performed in a blinded manner. This approach increases sensitivity for mild or subjectively reported changes but lacks standardization and may therefore overestimate less severe events. Moreover, severity grading was not possible, and comparison with PASI-based studies is only partially feasible. Finally, the observation window was limited to the three months following complete glucocorticoid discontinuation. Consequently, cutaneous deterioration occurring during glucocorticoid tapering or beyond the three-month follow-up period was not captured.

## 5. Conclusions

The present retrospective cohort study of 100 patients with psoriatic arthritis showed that documented cutaneous deterioration within three months after glucocorticoid discontinuation occurred in a minority of patients and was predominantly mild; erythrodermic or generalized pustular courses were not observed. No statistically significant association between cumulative glucocorticoid exposure and cutaneous deterioration was detected. These findings are consistent with recent systematic reviews by Vincken et al. (2022) and Long et al. (2022) as well as the PsA-specific cohort study by Bayrak and Aktas (2024) [7,16,24]. However, given the retrospective design and the absence of a control group, no conclusions can be drawn regarding a causal relationship between glucocorticoid discontinuation and subsequent cutaneous deterioration. Prospective controlled studies incorporating standardized assessments of skin involvement and tapering regimens are warranted to further clarify this association.

## Figures and Tables

**Figure 1:**
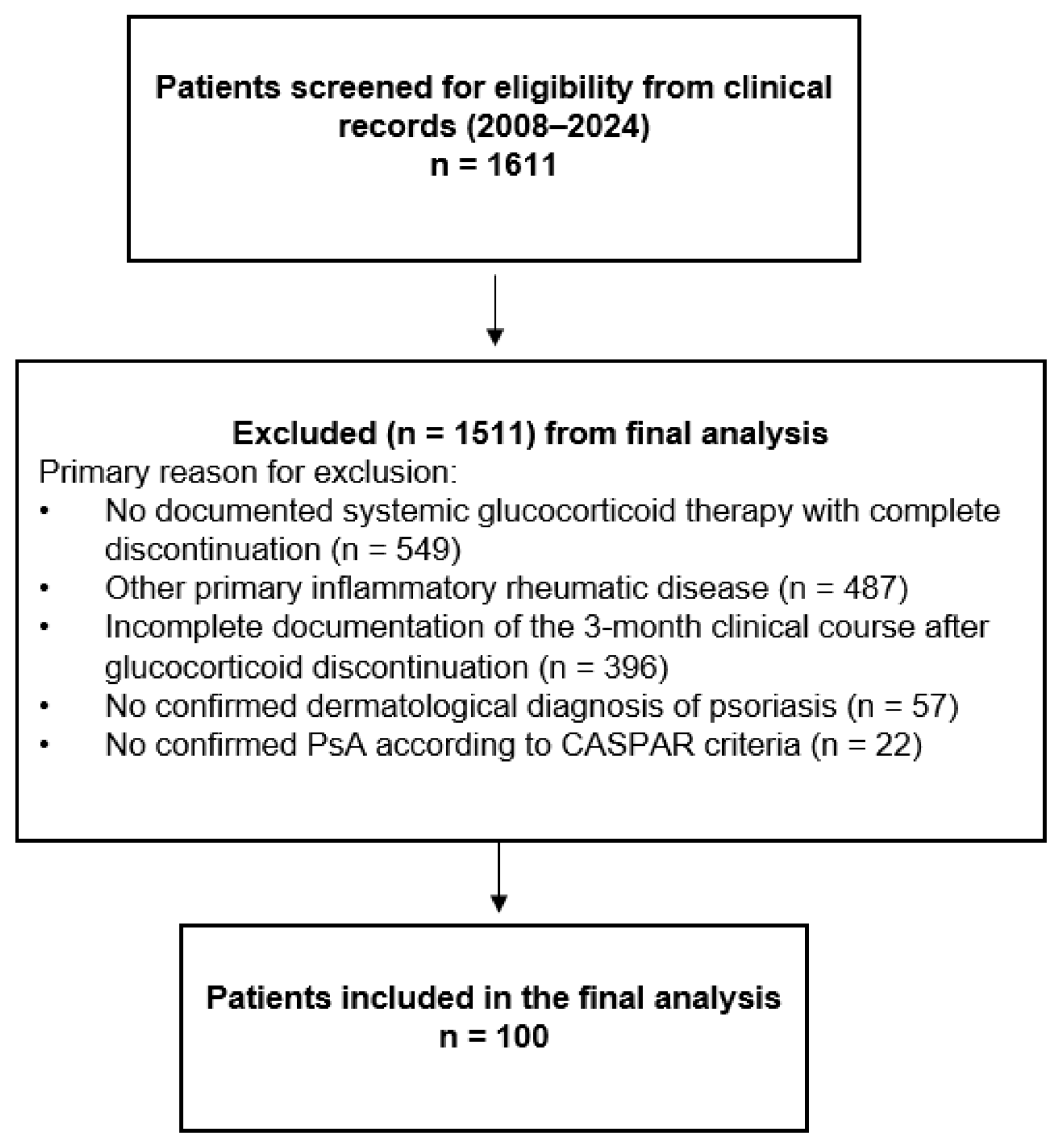
Flow chart showing the selection of 100 patients with psoriatic arthritis from 1611 screened patients, with inclusion and exclusion criteria applied at each step.

**Table 1:** Baseline demographic, clinical, and therapy-related characteristics of the study cohort (*n* = 100).

Variable	Total (*n* = 100)
Demographics	
Age at index visit, years	53.5 ± 13.6
Sex	
male, *n* (%)	60 (60.0%)
female, *n* (%)	40 (40.0%)
BMI, kg/m^2^	27.0 [25.1–30.8]
Type of stay	
inpatient, *n* (%)	58 (58.0%)
outpatient, *n* (%)	42 (42.0%)
Smoker, *n* (%)	12 (12.0%)
Comorbidities	
Coronary heart disease, *n* (%)	11 (11.0%)
Diabetes mellitus, *n* (%)	11 (11.0%)
Arterial hypertension, *n* (%)	36 (36.0%)
Hyperlipidaemia, *n* (%)	6 (6.0%)
Inflammatory bowel disease, *n* (%)	2 (2.0%)
Renal insufficiency, *n* (%)	6 (6.0%)
Dermatological diagnosis	
Psoriasis vulgaris, *n* (%)	89 (89.0%)
Pustular psoriasis, *n* (%)	9 (9.0%)
Erythrodermic psoriasis, *n* (%)	0 (0.0%)
Nail psoriasis, *n* (%)	2 (2.0%)
Rheumatic manifestation	
Monoarthritis, *n* (%)	25 (25.0%)
Oligoarthritis, *n* (%)	35 (35.0%)
Polyarthritis, *n* (%)	40 (40.0%)
Axial involvement, *n* (%)	17 (17.0%)
Enthesitis, *n* (%)	7 (7.0%)
Dactylitis, *n* (%)	16 (16.0%)
Active cutaneous involvement at presentation, *n* (%)	65 (65.0%)
Nail involvement at presentation, *n* (%)	14 (14.0%)
Therapy	
Antirheumatic therapy at the index visit	
NSAIDs, *n* (%)	13 (13.0%)
Conventional DMARDs, *n* (%)	61 (61.0%)
Biological DMARDs, *n* (%)	24 (24.0%)
JAK-Inhibitors, *n* (%)	2 (2.0%)
Methotrexate add-on therapy, *n* (%) or other non-MTX systemic therapy, *n* (%)	14 (14.0%)
Laboratory and disease activity	
CRP, mg/L	9.2 [2.9–21.9]
Glucocorticoid therapy	
Previous systemic glucocorticoid therapy, *n* (%)	52 (52.0%)
Intra-articular glucocorticoid injection at the index visit, *n* (%)	18 (18.0%)
Median prednisolone-equivalent starting dose, mg/day	12.5 [6.0–20.0]
Duration of therapy, weeks	16.0 [10.00–50.25]
Cumulative dose, mg	730 [354.38–1892.25]

Data are presented as *n* (%), mean ± standard deviation, or median [25th–75th percentile]. The number of analysed cases varies by variable due to missing values. Abbreviations: BMI, body mass index; CRP, C-reactive protein; ESR, erythrocyte sedimentation rate; CHD, coronary heart disease; IBD, inflammatory bowel disease. Hyperlipidaemia is defined as a physician-documented diagnosis of hypercholesterolaemia, hypertriglyceridaemia, or mixed hyperlipidaemia. Therapy: conventional DMARDs include methotrexate, leflunomide, and sulfasalazine. Biologics include TNF inhibitors (infliximab, adalimumab, golimumab, certolizumab, etanercept), the IL-12/23 inhibitor (ustekinumab), IL-17 inhibitors (secukinumab, ixekizumab), IL-23 inhibitors (guselkumab, risankizumab, tildrakizumab), and abatacept. Janus Kinase (JAK) inhibitors (tofacitinib, upadacitinib, filgotinib) are reported as a separate category. Reference range: CRP < 5 mg/L; ESR < 20 mm/h.

**Table 2:** Comparison of clinical features between patients with and without cutaneous deterioration following glucocorticoid discontinuation.

Variable	No Deterioration (*n* = 90)	Deterioration (*n* = 10)	*p*-Value
Age at index visit, years	54.1 ± 13.3	48.0 ± 15.7	0.18
Sex			0.084
female, *n* (%)	33 (36.7%)	7 (70.0%)	
male, *n* (%)	57 (63.3%)	3 (30.0%)	
BMI, kg/m^2^	27.1 [24.9–30.9]	26.1 [24.8–28.5]	0.296
Smoking status			1.000
Smoker, *n* (%)	11 (12.2%)	1 (10.0%)	
Non-smoker, *n* (%)	79 (87.8%)	9 (90.0%)	
Coronary heart disease			1.000
yes, *n* (%)	10 (11.1%)	1 (10.0%)	
no, *n* (%)	80 (88.9%)	9 (90.0%)	
Diabetes mellitus			0.302
yes, *n* (%)	9 (10.0%)	2 (20.0%)	
no, *n* (%)	81 (90.0%)	8 (80.0%)	
Arterial hypertension			1.000
yes, *n* (%)	33 (36.7%)	3 (30.0%)	
no, *n* (%)	57 (63.3%)	7 (70.0%)	
Hyperlipidaemia			1.000
yes, *n* (%)	6 (6.7%)	0 (0.0%)	
no, *n* (%)	84 (93.3%)	10 (100.0%)	
Inflammatory bowel disease			0.191
yes, *n* (%)	1 (1.1%)	1 (10.0%)	
no, *n* (%)	89 (98.9%)	9 (90.0%)	
Renal insufficiency			0.478
yes, *n* (%)	5 (5.6%)	1 (10.0%)	
no, *n* (%)	85 (94.4%)	9 (90.0%)	
Arthritis type			1.000
Monoarthritis, *n* (%)	23 (25.6%)	2 (20.0%)	
Oligoarthritis, *n* (%)	31 (34.4%)	4 (40.0%)	
Polyarthritis, *n* (%)	36 (40.0%)	4 (40.0%)	
Axial manifestation			0.064
yes, *n* (%)	13 (14.4%)	4 (40.0%)	
no, *n* (%)	77 (85.6%)	6 (60.0%)	
Enthesitis			1.000
yes, *n* (%)	7 (7.8%)	0 (0.0%)	
no, *n* (%)	83 (92.2%)	10 (100.0%)	
Dactylitis			1.000
yes, *n* (%)	15 (16.7%)	1 (10.0%)	
no, *n* (%)	75 (83.3%)	9 (90.0%)	
Active cutaneous involvement at presentation			1.000
yes, *n* (%)	58 (64.4%)	7 (70.0%)	
no, *n* (%)	32 (35.6%)	3 (30.0%)	
Nail involvement at presentation			0.628
yes, *n* (%)	12 (13.3%)	2 (20.0%)	
no, *n* (%)	78 (86.7%)	8 (80.0%)	
Background antirheumatic therapy			0.456
NSAIDs, *n* (%)	13 (14.4%)	0 (0.0%)	
Conventional DMARDs, *n* (%)	55 (61.1%)	6 (60.0%)	
Biological DMARDs, *n* (%)	20 (22.2%)	4 (40.0%)	
JAK-Inhibitors, *n* (%)	2 (2.2%)	0 (0.0%)	
Other, *n* (%)	0 (0.0%)	0 (0.0%)	
Methotrexate add-on therapy			0.628
yes, *n* (%)	12 (13.3%)	2 (20.0%)	
no, *n* (%)	78 (86.7%)	8 (80.0%)	
CRP, mg/L	9.43 [2.7–22.8]	8.05 [3.1–29.0]	0.909
GC starting dose, mg	12.0 [5.0–20.0]	15.0 [6.9–20.0]	0.727
GC duration, weeks	16.00 [10.0–53.0]	24.50 [14.0–45.8]	0.542
GC cumulative dose, mg	712.2 [351.9–1945.5]	1052 [393.5–1478.2]	0.904

Data are presented as mean ± standard deviation (SD), median [interquartile range (IQR)], or absolute frequencies n (%). For the comparison between groups, the chi-square test or Fisher’s exact test was used for categorical variables and the Mann–Whitney U test for continuous variables. A *p*-value < 0.05 was defined as statistically significant. Abbreviations: GC, glucocorticoid; BMI, body mass index; CRP, C-reactive protein; DMARDs, disease-modifying antirheumatic drugs; JAK, Janus kinase.

## Data Availability

The data presented in this study are available on request from the corresponding author due to privacy and ethical restrictions.
